# Identification of novel small ncRNAs in pollen of tomato

**DOI:** 10.1186/s12864-015-1901-x

**Published:** 2015-09-18

**Authors:** Kamila Lucia Bokszczanin, Nicolas Krezdorn, Sotirios Fragkostefanakis, Sören Müller, Lukas Rycak, Yuanyuan Chen, Klaus Hoffmeier, Jutta Kreutz, Marine J. Paupière, Palak Chaturvedi, Rina Iannacone, Florian Müller, Hamed Bostan, Maria Luisa Chiusano, Klaus-Dieter Scharf, Björn Rotter, Enrico Schleiff, Peter Winter, SPOT-ITN Consortium (Solanaceae Pollen Thermotolerance Initial Training Network Consortium)SPOT-ITN Consortium (Solanaceae Pollen Thermotolerance Initial Training Network Consortium)

**Affiliations:** GenXPro GmbH, Frankfurt am Main, Germany; Cluster of Excellence Frankfurt, Centre of Membrane Proteomics, Department of Biosciences, Goethe University, Frankfurt am Main, Germany; Department of Plant Breeding, Wageningen University and Research Centre, Wageningen, The Netherlands; Department for Molecular Systems Biology, University of Vienna, Vienna, Austria; ALSIA Research Center Metapontum Agrobios Metaponto (MT), Metaponto, Italy; Department of Molecular Plant Physiology, Institute for Water and Wetland Research, Radboud University Nijmegen, Nijmegen, The Netherlands; Department of Agricultural Sciences, University of Naples Federico II, Via Università 100, 80055 Portici, Italy

**Keywords:** sncRNA-seq, Tomato, Tetrad stage, Post-meiotic stage, Mature pollen, Heat stress, omiRas, miRANDA, tRNA, snoRNA

## Abstract

**Background:**

The unprecedented role of sncRNAs in the regulation of pollen biogenesis on both transcriptional and epigenetic levels has been experimentally proven. However, little is known about their global regulation, especially under stress conditions. We used tomato pollen in order to identify pollen stage-specific sncRNAs and their target mRNAs. We further deployed elevated temperatures to discern stress responsive sncRNAs. For this purpose high throughput sncRNA-sequencing as well as Massive Analysis of cDNA Ends (MACE) were performed for three-replicated sncRNAs libraries derived from tomato tetrad, post-meiotic, and mature pollen under control and heat stress conditions.

**Results:**

Using the omiRas analysis pipeline we identified known and predicted novel miRNAs as well as sncRNAs from other classes, responsive or not to heat. Differential expression analysis revealed that post-meiotic and mature pollen react most strongly by regulation of the expression of coding and non-coding genomic regions in response to heat. To gain insight to the function of these miRNAs, we predicted targets and annotated them to Gene Ontology terms. This approach revealed that most of them belong to protein binding, transcription, and Serine/Threonine kinase activity GO categories. Beside miRNAs, we observed differential expression of both tRNAs and snoRNAs in tetrad, post-meiotic, and mature pollen when comparing normal and heat stress conditions.

**Conclusions:**

Thus, we describe a global spectrum of sncRNAs expressed in pollen as well as unveiled those which are regulated at specific time-points during pollen biogenesis. We integrated the small RNAs into the regulatory network of tomato heat stress response in pollen.

**Electronic supplementary material:**

The online version of this article (doi:10.1186/s12864-015-1901-x) contains supplementary material, which is available to authorized users.

## Background

Non-coding RNAs (ncRNAs), derived from intergenic or intronic regions, antisense strands of protein-coding genes as well as pseudogenes, are essential components of various plant molecular machineries. According to their size, they are classified as small ncRNAs (sncRNAs) (<40 nt) and long ncRNAs (lncRNAs) (>200 nt), both involved in the transcriptional and posttranscriptional regulation of gene expression by modulation of RNA stability and translation under physiological and stress conditions [1–5].

In plants, knowledge regarding the biogenesis and mechanisms of action of sncRNA has been mainly gained through studies in *Arabidopsis* [6], but the introduction of high-throughput next generation sequencing (NGS) technologies has facilitated the rapid identification of small RNAs in different species including agriculturally important crops [7–10]. For instance pollen microRNAs (miRNAs) have been identified so far in loblolly (*Pinus taeda*) [11], cabbage (*Brassica campestris*) [12], rice (*Oryza sativa*) [9], and maize (*Zea* mays) [10].

We aimed at identifying sncRNAs including miRNAs, transfer RNAs (tRNAs), as well as small nucleolar RNAs (snoRNAs) expressed in the male germ cells of tomato (*Solanum lycopersicum*) – a model plant attributed to multiple expedient traits like relatively small, diploid, and almost fully sequenced and annotated genome [13, 14]. Because pollen has been proven to be the most sensitive plant tissue under heat conditions [15–17], we deployed elevated temperature conditions before harvesting the material to identify sncRNAs which are temperature stress-responsive. In here, we focus on the comparison of different developmental stages, based on the knowledge that earlier stages of pollen development (tetrads, early microsporogenesis) show higher sensitivity compared to more advanced stages (mature pollen). The meiotic phase of pollen development has been reported as an exceptionally thermosensitive stage of the reproductive process for a number of species (e.g., *Phaseolus vulgaris, Solanum lycopersicum, Vigna unguiculata*, *Capsicum annuum*) (Sato et al. 2002).

The important role of sncRNAs in the regulation of pollen biogenesis on both transcriptional and epigenetic levels has been well documented [18, 19]. It is considered that siRNAs and DNA methylation machinery play a part in setting up unique epigenetic landscapes in gametes, presumably contributing to their different fates and functions during fertilization. Indeed specific sncRNAs were identified in *Arabidopsis* male germ cells, therein the new class of Decrease in DNA methylation 1 (DDM1)-dependent 21 bp siRNAs produced from transposable elements (TEs) in the vegetative nucleus and DNA methyltransferase domains rearranged methylase 2 (DRM2)-dependent 24 bp siRNAs. In both microspores and germ cells CHH methylation is lost from retrotransposons due to down-regulation of DRM2, and subsequently de novo restored in the vegetative nucleus by DRM2-guided 24 bp siRNAs [19]. In the vegetative cell of microspores DDM1 inhibition-based retrotransposon activation leads to generation of 21 bp siRNAs which are subsequently translocated from the vegetative nucleus into the sperm cells to reinforce TE silencing [18]. Just recently it has been shown that miRNAs trigger widespread epigenetically activated siRNAs from transposons in *Arabidopsis* [20]. Expression data from both rice and *Arabidopsis* show that normally silenced TEs and neighboring sequences become transcribed during meiotic prophase [21, 22], pointing to a partial release of epigenetic repression. Interestingly, TE transcripts are not over-represented in the microspore transcriptome [23] indicating that this phase of activation is temporary and that epigenetic silencing pathway, presumably involving the RNA-dependent DNA methylation (RdDM), is restored post-meiosis [24].

High throughput approaches in combination with sncRNA-targeted studies enabled identification of sncRNAs involved in various stress responses, including heat [25–28]. Heat stress response is characterized by transcriptional reprogramming and preferential translation of genes mainly involved in maintenance of protein homeostasis, like HSPs (heat shock proteins). While the general regulatory network at protein level manifested by HSPs and heat shock transcription factors (HSFs) is currently explored at molecular level [29], the information on tissue specific reactions and on the importance of post-transcriptional and epigenetic mechanisms is still just emerging [30]. First findings showed that the RdDM pathway is required for basal heat tolerance in *A. thaliana*. Plants deficient in both DNA-directed RNA polymerases IV and V subunit 2 (NRPD2), the common second-largest subunit of RNA polymerases IV and V, and in the Rpd3-type histone deacetylase 6 (HDA6) were hypersensitive to heat exposure [31]. Further members of the HSF gene family, the major regulators of heat stress response, have been shown to be involved in a regulatory cascade in which central components are under the control of miRNAs. In *Arabidopsis* essential regulatory loop for thermotolerance has been revealed to comprise two Hsfs, miR398, and its target genes CSD1, CSD2 and CCS [30, 32]. HsfA1b and HsfA7b are responsible for the induction of miR398 under heat stress conditions, which then facilitates the degradation of CSD1, CSD2 and CCS to increase thermotolerance [30, 33]. Similarly, an *Arabidopsis* trans-acting siRNA precursor controls two HSF-regulated genes named Heat-Induced TAS1 Target 1 and 2 (HTT1 and 2), whose upregulation during heat stress increases plant thermotolerance [10].

Most of investigations utilizing high throughput NGS for the qualitative and quantitative determination of sncRNA transcripts have focused on miRNAs and siRNAs. However, other species like tRNAs and snoRNAs play key roles in plant stresses as well. For example, tRNAs are important for the transcriptional regulation under stress conditions as well as during the recovery phase. Full-length tRNAs are key for efficient and accurate protein translation. To be fully active, tRNAs need to be heavily modified post-transcriptionally [34]. However multiple groups have cloned and sequenced shorter tRNA fragments (tRFs) [35], which so far have been classified as fragments generated by cleavage at the anticodon loop to produce longer RNA species (~35 nt) called tRNA halves, and fragments of ~20 nt (often corresponding to a cleavage in the D or T loops), conspicuously similar to the size of siRNAs and miRNAs [36]. Therefore, it is hypothesized that the competition of tRNA fragments with post-transcriptional gene silencing (PTGS) would allow the translation of mRNAs important for the recovery from stress-induced cellular damage [37].

Here we describe the complete tomato pollen sncRNAome including miRNAs, tRNAs, and snoRNAs and show how these are affected by heat stress in different stages of the pollen development. Utilizing the omiRas web server together with plant-specific miRNA criteria for the identification of novel miRNA species we identify previously unknown miRNAs responding to the applied stress conditions in the post-meiotic stage and mature pollen. Targets of these novel miRNAs were determined by *in silico* prediction while their negatively correlated expression measured using MACE, a high-throughput expression profiling technique. Some of the targets have not been related till now to heat stress response but their predicted functions indicate possible involvement in thermotolerance as well as pollen development. Gene Ontology enrichment analysis revealed that most target genes of all expressed miRNAs were significantly enriched in protein binding, transcription, and Serine/Threonine kinase activity GO terms. Analysis of the individual tRNAs expression revealed that those corresponding to specific amino acids were affected more than others. This observation is of particular interest, considering the importance of tRNAs in protein synthesis under physiological and stress conditions.

Results of miRNAs and their targets identified in this study are additionally organized in an online database with a web interface (http://cab.unina.it/mirna-pollen/) giving the user a possibility to investigate expression profiles and the miRNA target GO annotation, while cross-linking to a *Gbrowse* Genome browser enables further genome wide investigations and comparative genomics.

## Methods

### Plant material and treatments

Tomato plants (*Solanum lycopersicum* cv. Red Setter) were grown under controlled conditions in the glasshouse facility of ALSIA – Research Center Metapontum Agrobios (Metaponto, Italy). Red Setter is a bush type tomato originated from the USA and has been described as resistant to *Verticillium* and *Fusarium*. However, its response to high temperatures is unknown from the literature. Growth conditions included a 12 hour light–dark cycle with a day temperature of 24-26 °C and night temperature of 18-20 °C, and relative humidity at 65-70 %. The daily solar radiation in the greenhouse was supplemented to at least 190 μmol photons m^−2^ s^−1^ from a series of high-pressure sodium lamps 400 W SON-T (Philips Electronics N.V. Amsterdam, Netherlands) after the appearance of the first inflorescence. During the first 6 weeks of development, plants were watered every two days and subsequently every day.

The short-term heat stress treatment and collection of pollen samples were performed according to the protocol developed by the SPOT-ITN consortium. For heat treatment plants were transferred in a preheated growth chamber and exposed to 38 °C for 1 h (Additional file 1: Figure S1) under artificial light (Photosynthetically active radiation (PAR) = 85,927 ± 4,7 μmol photons m^−2^s^−1^). The temperature was then gradually decreased to 25 °C within 30 minutes and plants were allowed to recover for an additional hour at 25 °C. Untreated plants were kept in the growth chamber for the same time period at control temperature of 25 °C. In a preliminary experiment (data not shown) the tomato flower bud size has been correlated with three stages of pollen development, using DAPI (4, 6-diamidino-2-phenylindole) staining as shown by Chaturvedi et al. (38). Flower material was harvested 1.5 h after HS and immediately collected in the containers kept on ice. Control samples were harvested from a parallel set of non‐stressed plants while performing the HS treatments. Flower buds were sorted according to the corresponding stages as follow: flower buds of 4–6 mm in length for tetrad (T) (meiotic stage/microspore mother cell), 6–8 mm in length for post-meiotic (PM) stage (microspores), and =/> 10 mm in length for mature (M) (bicellular pollen) pollen grains (Additional file 1: Figure S1).

Sepals from the flower buds of tetrad stage and sepals and petals from the flower buds of post‐meiotic and mature stages were gently removed using forceps. Additionally stigma and the style from the flower buds of post‐meiotic and mature stages were excised in order to avoid the contamination by parts of the gynoecium during pollen preparation. Immediately after removing aforementioned parts of the flower buds, released anthers were immersed in germination solution [KNO_3_ (1 mM); Ca(NO_3_)_2_ (3 mM); MgSO_4_ (0,8 mM), H_3_BO_3_ (1,6 mM)] kept on ice prior pollen isolation and purification.

Pollen was isolated from anthers by squeezing the stamens with a pipette tip 200 μl in the germination solution, followed by vortexing for 15 seconds. To ensure that the material was free of contaminants from other anther tissues, isolated pollen was passed through gauze cloth, washed twice with germination solution, and centrifuged at 4 °C for 2 min at 100 g. The supernatant was discarded, while pollen cell pellet re‐suspended in 200 μl of germination solution, followed by centrifugation at 100 g for 2 min at 4 °C. The supernatant was discarded and pollen samples were stored in the liquid nitrogen. Prior to first centrifugation the pollen stage and purity were confirmed by staining with 1 μg mL^−1^ DAPI and visualized under a fluorescent microscope [38] (Additional file 1: Figure S1).

Control and heat-stressed pollen samples were collected from three independent experiments performed during three consecutive days. Samples derived from one day were treated as biological replica.

### RNA isolation

Frozen samples were disrupted and homogenized using a TissueLyser (Qiagen) and RNA was isolated with NucleoSpin miRNA kit (Macherey-Nagel) in two fractions (small RNA < 200 nt and large RNA > 200 nt) according to manufacturer’s protocol.

### sncRNA library preparation

sncRNA libraries were prepared using the proprietary TrueQuant technology for elimination of PCR bias [39]. Briefly, modified 3′ and 5′ adapters (TrueQuant, GenXPro) were successive ligated to small RNA (<200 nt) using T4 RNA Ligase 2 and T4 RNA Ligase 1 (NEB), respectively. Adapter-ligated RNA was reverse transcribed with SuperScript III (Life Technologies) and amplified by PCR with KAPA HiFi Hot-Start Polymerase (KAPA Biosystems). Amplified libraries were size-selected by polyacrylamide gel electrophoresis and sequenced with HiSeq2000 (Illumina).

### sncRNA-seq data processing

The Illumina-derived sequence reads were pre-processed with an in-house analysis pipeline [39]. Briefly, libraries were sorted according to their respective index, followed by elimination of PCR-derived tags identified by TrueQuant technology. Both sequencing adapter and cDNA synthesis primers were removed in an additional quality filtering step. To investigate the functions of the different fractions we divided all samples into six parts according to their size and the abundance. We separated them according to the size into 22 nt, 24 nt and ‘others’ reads including all reads besides 22 nt and 24 nt with a minimum length of 20 nt. Further reads were sorted according to their abundance into singletons i.e. reads whose sequence occurs exactly once in a given sequencing library and non-singletons. After quantification of ncRNAs in each library, the data were combined to an expression matrix and processed with DESeq in omiRas webtool for annotation and differential expression of sncRNAs. Three pairwise comparisons were analyzed for three pollen stages between control and heat stress conditions.

### qPCR validation of sncRNAs

Primers for selected sncRNAs were custom designed using miRprimer software (http://sourceforge.net/projects/mirprimer). Sequences of all primers are provided in Additional file 2: Table S5. Total RNAs were reverse transcribed to cDNA using Universal cDNA Synthesis Kit II (Exiqon). PCR amplifications were performed using Fast SYBR Green Master Mix (Life Technologies) on a StepOne Real-Time PCR System (Applied Biosystems). All qPCR results were normalized based on 5S rRNA expression in each sample. Fold changes between samples were determined using the ΔΔCt method. Statistical analyses were performed with GraphPad Prism 6 (GraphPad Software) with sample size N = 3. Data is presented as mean + Standard Error of Mean (SEM).

### Annotation, differential expression, and novel miRNAs predictions

FastQ files from sncRNA-seq experiments were submitted to the omiRas web server [40, 41]. For miRNA analysis we used miRBase [42, 43], Tomato Genomic Resources Database [44, 45], Tomato Functional Genomics Database [46], and Rfam database [47, 48]. For tRNAs we deployed the SolGenomics ITAG2.4 database and for snoRNAs the Rfam database. snoRNAs from Rfam were mapped to the tomato genome.

Tags mapped to the exonic regions of protein coding genes were excluded from further analysis. Tags overlapping introns or intergenic regions not present in the aforementioned databases were used for the prediction of novel miRNAs with miRDeep-P [49] implemented in the omiRas web server.

For the prediction of novel miRNAs the following miRDeep-P criteria were used: i) tags longer than 15 nt which could be mapped to the tomato ITAG2.4 genome, ii) sliding window size for the RNA secondary structure prediction – 270 nt, iii) secondary structure together with mapped reads processed according to plant scoring system – maximal value of the log-odds score from the minimum free energy based on the Gumbel distribution [49] and, iv) plant-specific criteria for miRNAs [50]. Secondary structure prediction and visualization was carried out by RNAfold [51] provided in the ViennaRNA Package 2.0 [52] according to RNA parameters described [53]. As a result novel putative miRNAs have been identified along with their stem-looped precursors and location on the tomato ITAG2.4 genome.

As novel miRNAs we considered predicted mature miRNAs longer than 20 nt and following plant-specific criteria: (1) The miRNA and miRNA* are derived from opposite stem-arms such that they form a duplex with two nucleotide, 3’ overhangs (an outcome of the Dicer cleavage); (2) base-pairing between the miRNA and the other arm of the hairpin, which includes the miRNA*, is extensive such that there are typically four or fewer mismatched miRNA bases; and (3) asymmetric bulges are minimal in size (one or two bases) and frequency (typically one or less), especially within the miRNA/miRNA* duplex [50].

The differential expression of sncRNAs between control and stressed samples for each developmental stage was calculated by DESeq (R package version 1.16.0) [54] considering the statistical power of the three biological replicates per sample and log2 fold change. RNAs with a corrected p-value (FDR) below 0.05 were considered significantly differentially expressed.

### miRNA target prediction

The software miRanda version: 3.3a [55] was used to predict the targets for the sncRNA. As input we took the data described in “raw data processing”. The consensus sequences between the MACE reads and the reference of MACE were taken as potential target sequences. We limited the results for one sncRNA to the 10 best results/targets given by miRanda. The results were filtered according to the alignment score and the energy generated as standard parameters by miRanda. Approximately six nucleotides starting at position two from the 5’end of the mature sequence are considered to be particularly important for the target recognition [56]. The miRanda algorithm takes into account sequence-matching to assess whether two sequences are complementary and possibly bind and free energy calculation (thermodynamics) to estimate the energetics of this physical interaction [55]. We connected differential expression of both miRNAs and their potential targets to increase the likelihood of biological relevance. Target differential expression was originated from genome-wide gene expression profiling performed by MACE (Massive Analysis of cDNA Ends).

### MACE library preparation

MACE libraries were prepared using the proprietary TrueQuant technology for elimination of PCR bias [39]. Briefly, poly-adenylated RNA was extracted with Dynabeads mRNA Purification kit (Life Technologies) from large RNA (>200 nt) and reverse transcribed with SuperScript Double-Stranded cDNA Synthesis Kit (Life Technologies) using biotinylated poly(dT) primers. cDNA was fragmented with Bioruptor (Diagenode) to an average size of 250 bp. Biotinylated cDNA ends were captured by Dynabeads M-270 Streptavidin Beads (Life Technologies) and ligated with T4 DNA Ligase 1 (NEB) to modified adapters (TrueQuant, GenXPro). The libraries were amplified by PCR with KAPA HiFi Hot-Start Polymerase (KAPA Biosystems), purified by Agencourt AMPure XP beads (Beckman Coulter) and sequenced with HiSeq2000 (Illumina).

### MACE data analysis

Four bioinformatical steps were performed. First, reads were mapped to the tomato genome (ITAG2.4) and annotated using public annotation files. Reads that mapped to genomic regions with no annotation were clustered. These clusters (consensus sequences) were annotated via BLASTX to the Uniprot database. Reads not mapped to the tomato genome were mapped to the tomato transcriptome (ITAG2.4) and annotated using public annotation files. In the last step reads that did not map to the genome or trancriptome were de-novo-assembled. The resulting contigs were annotated via BLASTX to the Uniprot database. The differential expression of mRNAs between control and stressed samples for each developmental stage was calculated by DESeq (R package version 1.16.0) [54] considering the statistical power of the three biological replicates per sample and log2 fold change. Transcripts with a corrected p-value (FDR) below 0.05 were considered significantly differentially expressed.

### Gene Ontology analysis

Gene Ontology analysis has been performed using STDGE2GO, a web based toolkit developed by GenXPro [57], which provides the interpretation and visualization of biological relations for high-throughput experimental results using the Gene Ontology (GO) System. GO categories for MACE data were annotated using the UniProt-GOA database (EBI) and information by ENSEMBL.

## Results

### High throughput sequencing results of tomato pollen sncRNA libraries

For the identification of heat stress related sncRNAs in tomato pollen we constructed libraries, using RNA isolated from three pollen developmental stages of tomato cv Red Setter plants kept under control and heat stress conditions (see Materials). Deep sequencing led to the generation of a total number of about 14 and 10 million raw reads for the three examined developmental stages originated from control and heat treated libraries, respectively (Table 1). We were able to map 92-98 % reads to the tomato genome with the lowest and the largest fraction being annotated to the libraries of heat-treated post-meiotic and mature pollen, respectively (Table 1). Reads were mapped to intergenic, ncRNA, intronic, and exonic regions of the tomato genome with 64-84 % mapped to the intergenic and only 2-5 % reads annotated to the exonic or intronic regions (Table 1).

**Table 1:** Illumina sncRNA-sequencing reads generated for tomato pollen libraries including the total number of sequenced reads and mapped to the tomato genome

	T_C	T_H	PM_C	PM_H	M_C	M_H
No of reads						
Total	6 018 023	4 189 102	4 826 761	3 217 179	3 241 022	3 330 945
Mapped	2 581 350	1 740 320	1 438 733	1 035 440	1 464 860	1 395 572
intergenic	2 153 220	1 453 333	1 028 934	667 284	1 270 017	1 397 557
intronic	90 055	59 209	53 201	34 803	76 544	79 921
Exonic	73 333	55 311	42 181	28 389	38 031	45 414

Intergenic, intronic, and exonic reads are a subpool of mapped reads. (*T* tetrad, *PM* post-meiotic, *M* mature, *C* control, *H* heat)

**Fig. 1:**
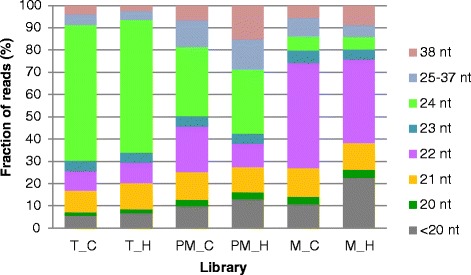
Global analysis of the dataset. The fraction of sncRNA Illumina sequencing reads with indicated length in three pollen libraries from control and heat conditions is shown. (T – tetrad, PM – post-meiotic, M – mature, C - control, H – heat stress)

**Fig. 2:**
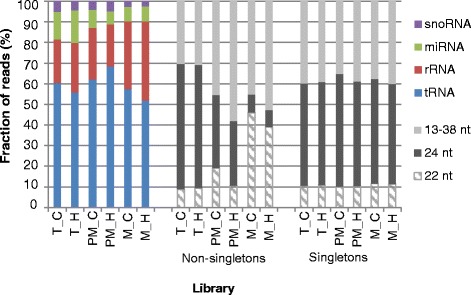
Detected sncRNAs. The fraction of Illumina sequencing reads mapped to tRNA, rRNA, miRNA, and snoRNA as well as the fraction of non-singletons and singletons with 22, 24, and 13–38 nt length in three tomato pollen libraries from control and heat conditions are shown. (T – tetrad, PM – post-meiotic, M – mature, C - control, H – heat)

Small RNAs (sRNAs) are 18–30 nt non-coding regulatory elements found in diverse organisms, which were initially identified as small double-stranded RNAs in *Caenorhabditis elegans* [58]. Among them, miRNAs and siRNAs are found to be very important riboregulators in plants [58]. miRNAs constitute small 20–24 nt in length RNA molecules [59–62]. In our data set the length of sncRNAs ranged from 13–38 nt (Fig. 1). The most abundant sncRNAs in tetrad and post-meiotic stages were 24 nt reads, while in the mature pollen 22 nt reads were the major fraction (Fig. 1). Generally heat stress did not affect the abundance of 22 and 24 nt sncRNAs in tetrads, but we observed a decrease of 22 nt reads in heat stressed post-meiotic and mature pollen samples (Fig. 2). Because sncRNAs of 22 nt and 24 nt in length have been found to play specific roles during pollen development in *Arabidopsis* in both vegetative and generative cells [18], remarkable changes in their numbers in the investigated libraries can point to time-dependent production and regulation in the course of pollen biogenesis.

54 % of all assigned reads represent singletons, while the remaining 46 % of all reads define non-singletons. However, the fraction of 22 and 24 nt singletons remained unchanged between the pollen stages in both control and stressed samples, whereas non-singletons were more abundant in tetrads and post-meiotic pollen in control compared to the heat stressed samples (Fig. 2).

In depth analysis of sncRNA revealed that we detected tRNA, rRNA, pre-miRNA, and snoRNA, with tRNAs constituting the largest fraction, accounting for more than 50 % of tags, while pre-miRNA accounted for 7-16 % in the different libraries (Fig. 2).

### Expression analysis of snoRNAs and tRNAs under heat stress

We found one and six heat-responsive snoRNAs in post-meiotic and mature pollen, respectively (Table 2), but we did not identify snoRNAs affected by heat stress in tetrad stage. Four of the snoRNAs were significantly up- and three down-regulated in response to heat stress (Table 2). Among them, U1 and U3 snoRNAs were induced by heat in mature pollen while U4 in post-meiotic pollen stages, indicating possible involvement in the regulatory changes caused by stress in specific stages of male gametophyte development.

**Table 2:** snoRNAs with different transcript abundance under normal and under heat stress condition in at least one pollen stage

snoRNA id	Library					
	Tetrad		Post-meiotic		Mature	
	log_2_fc	fdr	log_2_fc	fdr	log_2_fc	fdr
sly-snoRNA-snoR101	−0.10	1.00	−1.69	0.09	**-inf**	0.00
sly-snoRNA-U1	−0.44	0.89	1.03	0.69	**1.77**	0.00
sly-snoRNA-R24	−0.28	0.93	−0.33	1.00	**1.58**	0.02
sly-snoRNA-U3	0.08	1.00	0.33	1.00	**0.85**	0.03
sly-snoRNA-U4	−1.08	0.38	**2.21**	0.00	−0.04	0.81
sly-snoRNA-snoR31_Z110_Z27	−0.40	0.89	−0.32	1.00	**−2.56**	0.04
sly-snoRNA-snoR32_R81	−0.53	0.89	−0.17	1.00	**−3.33**	0.03

The significant fold change (fc) values (fdr < 0.05) are indicated in bold

Notes: *inf* infinitive

tRNAs are a multi-functional molecules involved in many processes of cellular metabolism [64]. Moreover, tRNA derived fragments are crucial in plant stress response [65]. The global analysis of the expression of tRNAs in the different pollen stages revealed that in tetrad the total abundance of Lys tRNAs is reduced upon heat stress application, while Ala- and Val tRNAs are enhanced (Table 3). In post-meiotic stages the abundance of Gln, Lys, Thr- and Val tRNAs are enhanced (Table 3). In mature pollen, we observed an enhanced level of Ala-, Ile-, Leu-, and Phe-tRNA, and a reduction of Glu-, Gln, Gly, Pro-, and Val-tRNA (Table 3).

**Table 3:** Number of amino acid-based clustered tRNA transcripts per million under control and heat in three pollen developmental stages and their change established under heat in %

Amino acid cluster	Tetrad			Post-meiotic			Mature		
	Control	Heat	Change (%)	Control	Heat	Change (%)	Control	Heat	Change (%)
Ala	15373	23781	**35.36**	22781	27054	15.79	19136	37796	**49.37**
Arg	13455	18817	28.49	22563	24781	8.95	14774	20454	27.77
Asp	9492	8109	−14.57	13944	17693	21.19	14857	10835	−27.07
Cys	1739	1841	5.55	3451	3366	−2.45	1937	1861	−3.90
Glu	27364	20351	−25.63	34189	48155	29.00	23705	7401	**−68.78**
Gln	4236	3146	−25.72	3855	7597	**49.25**	1870	1052	**−43.72**
Gly	24239	17803	−26.55	28225	39250	28.09	18381	7983	**−56.57**
His	5028	6630	24.16	7973	9038	11.78	4917	5254	6.41
Ile	932	1095	14.90	1081	1040	−3.79	2271	3679	**38.28**
Leu	2222	2576	13.74	2203	3042	27.58	2415	4007	**39.74**
Lys	2624	1828	**−30.33**	2828	5188	**45.50**	4279	3159	−26.18
Met	693	736	5.87	1406	1120	20.33	2378	2355	−0.96
Phe	411	421	2.44	435	527	17.40	966	1968	**50.91**
Pro	3154	2739	−13.17	6908	5037	−27.08	5687	2853	**−45.36**
Ser	4026	4814	16.36	5528	6281	11.98	5433	9943	23.02
Thr	2538	2381	−6.19	2506	4186	**40.15**	2445	1882	−2.86
Trp	245	175	−28.52	430	545	21.06	955	928	−16.83
Tyr	2000	2496	19.85	2965	3189	7.02	3153	3791	23.92
Val	2132	1323	**−37.92**	1619	3992	**59.45**	1791	1363	**−31.40**

(+% enhanced upon heat, −% reduced upon heat) changes of larger than 30 % are highlighted)

**Fig. 3:**
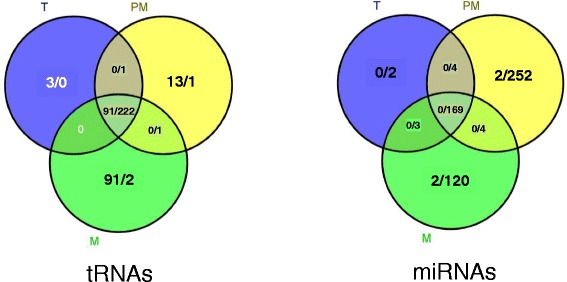
tRNAs and miRNAs distribution in pollen tissues. Venn diagram of heat responsive/not responsive tRNAs and miRNAs (fdr <0.05, log2 fold change) detected in three tomato pollen libraries (T – tetrad, PM – post-meiotic, M – mature, fdr – false discovery rate)

Prompted by the observed global change we analyzed the individual tRNAs. We observed the transcripts of 313 tRNAs common for all three pollen stages analyzed (Fig. 3). Among them the transcript level of 91 was altered after heat-stress application (Fig. 3). In addition, we identified transcripts of 112 tRNAs, heat-responsive or not, in at least one of the pollen stages analyzed, most of them in mature pollen (Fig. 3). Remarkably, of the tRNAs found in all pollen stages, three, 13, and 91 tRNAs were significantly either up- or down-regulated under heat in tetrad (Table 4), post-meiotic (Table 5), and mature pollen (Table 3), respectively.

**Table 4:** tRNAs in tetrads. Log 2 fold changes (log2fc) of significantly differentially expressed tRNAs (fdr < 0.05) are shown

AA	tRNA id	log2fc	log2fc
GLY	sly-tRNA-codon = CCC;Gly	−1.27	0.05
	sly-tRNA-codon = CCC;Gly-3	−1.23	0.04
	sly-tRNA-codon = CCC;Gly-4	−1.34	0.03

**Table 5:** tRNAs in post-meiotic pollen. Log 2 fold changes (log2fc) of significantly differentially expressed tRNAs (fdr < 0.05) are shown

AA	tRNA id	log2fc	fdr
ARG	sly-tRNA-codon = TCT;Arg	1.81	0.01
	sly-tRNA-codon = TCT;Arg-2	1.71	0.01
GLY	sly-tRNA-codon = CCC;Gly	1.42	0.05
	sly-tRNA-codon = GCC;Gly-5	1.69	0.01
LEU	sly-tRNA-codon = CAG;Leu	2.39	0.01
	sly-tRNA-codon = CAG;Leu-5	1.94	0.01
	sly-tRNA-codon = CAG;Leu-6	1.96	0.01
LYS	sly-tRNA-codon = CTT;Lys	2.18	0.01
	sly-tRNA-codon = CTT;Lys-2	1.48	0.02
	sly-tRNA-codon = CTT;Lys-4	1.6	0.01
VAL	sly-tRNA-codon = AAC;Val-3	1.91	0.01
	sly-tRNA-codon = AAC;Val-4	2.31	0.01
	sly-tRNA-codon = AAC;Val-6	1.85	0.01

**Fig. 4:**
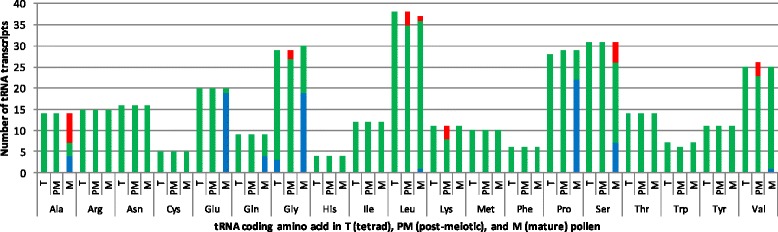
The amino-acid specific tRNA regulation. The number of amino acid-based clustered tRNAs significantly (fdr < 0.05) up- (red), down-regulated (blue) and not altered in expression (green) in three pollen libraries from control and heat conditions

The analysis of the individual tRNAs revealed that those corresponding to specific amino acids were affected more than others (Tables 4, 5 and 6). The majority of the Glu-, Gly- and Pro-coded tRNAs were specifically down-regulated by stress in mature stage (Fig. 4). Similarly, approximately half of the Ala-coded tRNAs were induced, while one third was reduced in expression (Fig. 4). This observation is of particular interest, considering the importance of tRNAs in protein synthesis under physiological and stress conditions.

**Table 6:** tRNAs in mature pollen. Log 2 fold changes (log2fc) of significantly differentially expressed tRNAs (fdr < 0.05) are shown

AA	tRNA id	log2fc	fdr	AA	tRNA id	log2fc	fdr
ALA	sly-tRNA-codon = AGC;Ala	1.33	0.01	GLY	sly-tRNA-codon = CCC;Gly	−2.43	0
	sly-tRNA-codon = TGC;Ala	1.23	0.03		sly-tRNA-codon = TCC;Gly	−0.99	0.04
	sly-tRNA-codon = AGC;Ala-2	1.31	0.01		sly-tRNA-codon = TCC;Gly-11	−1.03	0.04
	sly-tRNA-codon = TGC;Ala-2	1.3	0.02		sly-tRNA-codon = TCC;Gly-12	−1.25	0.01
	sly-tRNA-codon = AGC;Ala-3	1.31	0.01		sly-tRNA-codon = CCC;Gly-2	−2.44	0
	sly-tRNA-codon = TGC;Ala-4	1.3	0.02		sly-tRNA-codon = TCC;Gly-2	−1.09	0.02
	sly-tRNA-codon = TGC;Ala-5	1.3	0.02		sly-tRNA-codon = CCC;Gly-3	−2.36	0
	sly-tRNA-codon = AGC;Ala-5	1.14	0.03		sly-tRNA-codon = TCC;Gly-3	−1.02	0.04
	sly-tRNA-codon = AGC;Ala-6	1.32	0.01		sly-tRNA-codon = CCC;Gly-4	−2.39	0
	sly-tRNA-codon = TGC;Ala-6	1.29	0.02		sly-tRNA-codon = TCC;Gly-4	−1.38	0
	sly-tRNA-codon = TGC;Ala-7	1.3	0.02		sly-tRNA-codon = GCC;Gly-5	−0.85	0.03
GLU	sly-tRNA-codon = CTC;Glu	−1.67	0		sly-tRNA-codon = TCC;Gly-5	−1.04	0.03
	sly-tRNA-codon = TTC;Glu	−1.49	0		sly-tRNA-codon = GCC;Gly-6	−2.05	0
	sly-tRNA-codon = CTC;Glu-10	−2	0		sly-tRNA-codon = TCC;Gly-6	−1.04	0.04
	sly-tRNA-codon = TTC;Glu-11	−1.4	0		sly-tRNA-codon = TCC;Gly-7	−1.03	0.03
	sly-tRNA-codon = CTC;Glu-12	−1.99	0		sly-tRNA-codon = CCC;Gly-8	−1.39	0
	sly-tRNA-codon = CTC;Glu-2	−2.03	0		sly-tRNA-codon = TCC;Gly-8	−1.04	0.04
	sly-tRNA-codon = TTC;Glu-2	−1.46	0		sly-tRNA-codon = CCC;Gly-9	−2.19	0
	sly-tRNA-codon = CTC;Glu-3	−2.37	0		sly-tRNA-codon = TCC;Gly-9	−1.03	0.04
	sly-tRNA-codon = TTC;Glu-3	−2.12	0	SER	sly-tRNA-codon = GCT;Ser	1.96	0
	sly-tRNA-codon = CTC;Glu-4	−2.17	0		sly-tRNA-codon = TGA;Ser	1.28	0.02
	sly-tRNA-codon = TTC;Glu-4	−1.45	0		sly-tRNA-codon = TGA;Ser-12	1.3	0.02
	sly-tRNA-codon = CTC;Glu-5	−1.97	0		sly-tRNA-codon = TGA;Ser-13	1.3	0.02
	sly-tRNA-codon = TTC;Glu-5	−1.36	0		sly-tRNA-codon = GCT;Ser-2	1.67	0
	sly-tRNA-codon = CTC;Glu-6	−2.02	0		sly-tRNA-codon = TGA;Ser-2	1.31	0.01
	sly-tRNA-codon = TTC;Glu-6	−1.39	0		sly-tRNA-codon = TGA;Ser-3	1.55	0
	sly-tRNA-codon = CTC;Glu-7	−2	0		sly-tRNA-codon = GCT;Ser-3	1.54	0.01
	sly-tRNA-codon = TTC;Glu-7	−1.42	0		sly-tRNA-codon = GCT;Ser-4	1.91	0
	sly-tRNA-codon = TTC;Glu-8	−1.39	0		sly-tRNA-codon = TGA;Ser-4	1.63	0
	sly-tRNA-codon = TTC;Glu-9	−1.35	0.01		sly-tRNA-codon = GCT;Ser-6	1.35	0.03
GLN	sly-tRNA-codon = CTG;Gln	−1.73	0		sly-tRNA-codon = AGA;Ser	1.08	0.02
	sly-tRNA-codon = CTG;Gln-2	−1.26	0.01	PRO	sly-tRNA-codon = CGG;Pro	−1.81	0
	sly-tRNA-codon = CTG;Gln-3	−1.72	0		sly-tRNA-codon = AGG;Pro	−1.58	0
	sly-tRNA-codon = CTG;Gln-4	−2.33	0		sly-tRNA-codon = TGG;Pro-10	−1.92	0
LEU	sly-tRNA-codon = CAA;Leu-13	−1.16	0.04		sly-tRNA-codon = AGG;Pro-10	−1.6	0.01
	sly-tRNA-codon = CAG;Leu-5	1.4	0.04		sly-tRNA-codon = TGG;Pro-11	−1.92	0
	sly-tRNA-codon = CAA;Leu-8	−1.16	0.04		sly-tRNA-codon = AGG;Pro-12	−1.58	0.01
VAL	sly-tRNA-codon = AAC;Val-7	−1.04	0.04		sly-tRNA-codon = TGG;Pro-13	−1.94	0
PRO	sly-tRNA-codon = AGG;Pro-3	−1.52	0.01		sly-tRNA-codon = TGG;Pro-14	−1.92	0
	sly-tRNA-codon = AGG;Pro-4	−1.62	0.01		sly-tRNA-codon = CGG;Pro-14	−1.53	0
	sly-tRNA-codon = TGG;Pro-5	−1.67	0		sly-tRNA-codon = CGG;Pro-15	−1.54	0
	sly-tRNA-codon = AGG;Pro-6	−1.62	0.01		sly-tRNA-codon = TGG;Pro-2	−1.66	0
	sly-tRNA-codon = TGG;Pro-7	−1.96	0		sly-tRNA-codon = AGG;Pro-2	−1.53	0
	sly-tRNA-codon = CGG;Pro-8	−2.41	0		sly-tRNA-codon = CGG;Pro-3	−2.27	0
	sly-tRNA-codon = AGG;Pro-8	−1.57	0.01		sly-tRNA-codon = TGG;Pro-3	−1.7	0
	sly-tRNA-codon = TGG;Pro-9	−1.86	0

### Identification of tomato pollen miRNAs and their putative mRNA targets

We found 77 known, primarily identified either in leaf or tomato fruit, miRNAs constitutively expressed in tetrad and post-meiotic stage, and 76 in mature pollen (Additional file 3: Table S1). Among them, sly-miR-076-tgdb was expressed only in tetrad stage, while others were detectable in at least two stages (Additional file 3: Table S1). Further we found 169 known and novel miRNAs expressed in all pollen stages pointing to their role during whole process of pollen biogenesis (Fig. 3). None of the known tomato miRNAs from the omiRas data depository were identified as differentially expressed in response to HS in tomato pollen stages.

In order to identify new miRNAs expressed in pollen we employed miRDeep-P algorithm [49]. This led to the identification of 354 and 222 new putative miRNAs in post-meiotic (Additional file 4: Table S2) and mature pollen (Additional file 5: Table S3), with 254 and 122 specific to the respective pollen stages. We predicted 347 and 823 targets for 299 and 96 known and novel miRNAs, respectively (Additional file 6: Table S4).

**Fig. 5:**
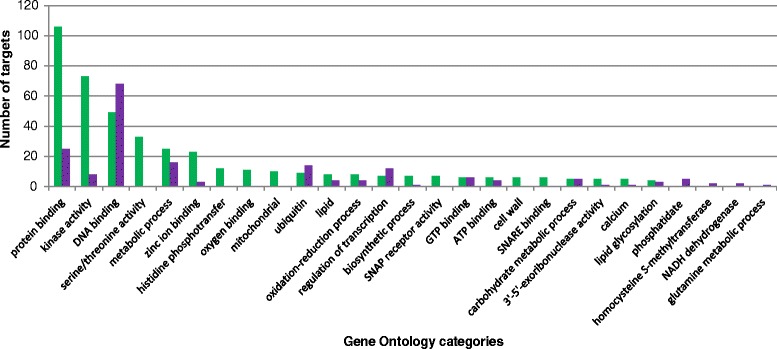
Gene Ontology annotation of miRNAs in pollen. Shown is the number of predicted targets in one gene ontology group of novel (green) and known tomato miRNAs (violet)

To gain insight into biological processes assigned to genes targeted by tomato pollen miRNAs we performed GO analysis (Fig. 5, Additional file 6: Table S4). We found 43 and 154 specific GO categories for known and novel miRNAs, respectively among which 45 were commonly found in both sets. Most of the transcripts have been annotated to protein binding, kinase activity, and DNA binding categories (Fig. 5). There were also targets annotated to oxidation-reduction, regulation of transcription, carbohydrate metabolic processes, ATP, and GTP binding. Specifically targets of novel miRNAs have been associated to Serine/Threonine kinase, histidine phosphotransfer, SNAP (Soluble NSF (N-ethylmaleimide-sensitive factor) Attachment Protein) receptor activity, and SNARE (SNAP Receptor) binding (Fig. 5), while targets of known miRNAs were specifically categorized to homocysteine S-methyltransferase and NADH dehydrogenase activities, and glutamine-related metabolic processes (Fig. 5).

### Identification of novel pollen miRNAs responsive to heat stress and their putative targets

**Fig. 6:**
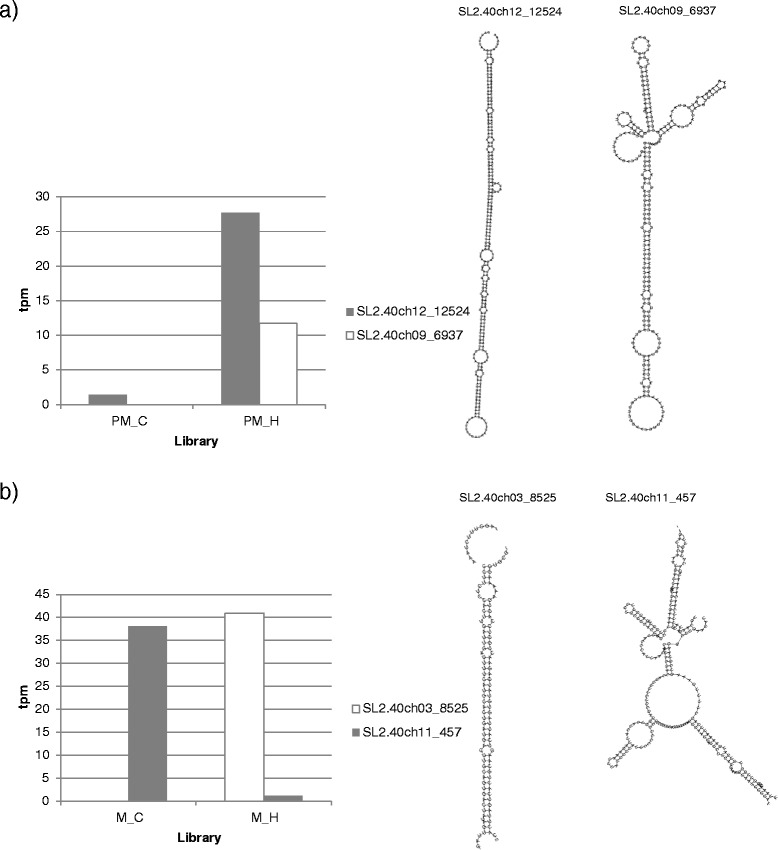
Four miRNAs with heat specific regulation in pollen. Transcripts per million (tpm) of novel miRNAs in (**a**) post-meiotic and (**b**) mature pollen libraries from control and heat conditions are shown. Mature sequences of these miRNAs are provided. (PM – post-meiotic, M – mature, C - control, H – heat)

The expression of most of the identified miRNAs did not change in response to heat stress. However, we found pollen stage-specific miRNAs differentially expressed in response to heat. Two novel 21 nt miRNAs predicted to be originated from tomato chromosome 9 and 12 (SL2.40ch09_6940; SL2.40ch12_12524) were identified in post-meiotic stage and both are up-regulated under heat. In mature pollen two 24 nt miRNAs mapped to the chromosome 3 and 11 (SL2.40ch03_8525; SL2.40ch11_457), one down- and the other up-regulated in response to heat stress (Fig. 6; Additional file 4: Table S2 and Additional file 5: Table S3). The secondary structures of the predicted precursor miRNAs was predicted by RNAfold [51] and presented in the Fig. 6.

We found that the newly identified miRNAs target all together 17 mRNAs in the post-meiotic stage and 14 mRNAs in the mature pollen (Table 7). None of the predicted targets showed statistically significant differential transcript abundance in response to heat. Only the gene coding for a peroxidase (Solyc01g007950) had a nearly 50 % reduction, which correlates to the 4-fold up-regulation of the SL2.40ch12_12524 miRNA (Fig. 6). However miRNAs are also involved in the regulation of translation efficiency [66].

**Table 7:** List of targets of differentially expressed novel miRNAs in post-meiotic and mature pollen libraries

	mR	Target ID	Target annotation	FE	Tpm-N	Tpm H	Log2	Fdr
post-meiotic pollen	SL2.40ch12_12524 (21 nt)	Solyc02g071750.2.1	Cyclic phosphodiesterase	−135.31 kcal/mol	374.45	759.93	1.02	0.24
		Solyc02g080670.2.1	Laccase-22 (Multicopper oxidase)		10.46	37.21	1.83	0.95
		Solyc07g054730.1.1	Wound-responsive protein		23.10	30.34	0.39	0.95
		Solyc01g007950.2.1	Peroxidase 1		1657.51	734.30	−1.17	0.95
		Solyc12g095990.1.1	ATP-dependent RNA helicase eIF4A		698.37	682.61	−0.03	1.00
		Solyc04g011720.2.1	Glucan endo-1 3-beta-glucosidase 5		21.02	19.86	−0.08	1.00
		Solyc12g020110.1.1	DNA repair protein rad5		29.93	27.59	−0.12	0.95
		Solyc02g067040.2.1	Unknown		17.07	13.79	−0.31	0.95
	SL2.40ch09_6940 (21 nt)	Solyc04g082050.2.1	Cellular retinaldehyde-binding	−84.1 kcal/mol	33.75	43.80	0.38	0.95
		Solyc03g033320.2.1	Prolyl 4-hydroxylase alpha		8.54	7.60	−0.17	0.98
		Solyc06g073690.2.1	Transducin family protein		0.59	1.50	1.36	1.00
		Solyc07g007890.2.1	CCR4-NOT transcription complex		93.87	122.73	0.39	0.95
		Solyc11g008990.1.1	Phage shock protein A PspA (PspA/IM30)		24.38	18.43	−0.40	0.95
		Solyc04g064490.2.1	Glycosyltransferase		188.92	148.32	−0.35	0.95
		Solyc02g092230.2.1	Adiponectin receptor 2		390.97	347.03	−0.17	0.99
		Solyc01g108650.2.1	Unknown		33.75	43.80	0.38	0.95
Mature pollen	SL2.40ch03_8525 (24 nt)	Solyc02g062570.2.1	Dolichyldiphosphatase 1	−56.10 kcal/mol	3.98	16.12	2.02	1.00
		Solyc06g084620.1.1	Pectinesterase		97384.21	103657.50	0.09	1.00
		Solyc02g090180.2.1	Oligopeptidase (Protease II)		9.94	4.22	−1.24	1.00
		Solyc07g014700.2.1	Dolichyl-diphosphooligosaccharide-protein glycosyltransferase		409.76	304.51	−0.43	1.00
		Solyc02g081010.1.1	Transcription factor jumonji		39.63	72.87	0.88	0.39
		Solyc03g093240.2.1	GTP-binding protein YqeH (GTP-binding protein HSR1-related)		2.73	1.43	−0.94	1.00
		Solyc09g011820.2.1	Unknown		6.34	13.52	1.09	1.00
	SL2.40ch11_457 (24 nt)	Solyc01g094190.2.1	dTDP-4-dehydrorhamnose reductase	−56.79 kcal/mol	52.69	47.62	−0.15	1.00
		Solyc01g090180.2.1	Extradiol ring-cleavage dioxygenase class III protein		5.83	3.78	−0.62	1.00
		Solyc01g087430.2.1	PHD zinc finger-containing protein		10.75	21.14	0.98	1.00
		Solyc10g077040.1.1	Magnesium-protoporphyrin IX monomethyl ester		26.81	19.03	−0.49	1.00
		Solyc06g084440.2.1	Nuclear protein localization 4 (NPL4)		187.57	191.29	0.03	1.00
		Solyc09g064860.2.1	Ubiquitin system component Cue		291.99	256.31	−0.19	1.00
		Solyc07g040980.2.1	Unknown		108.10	117.99	0.13	1.00

Given are the miRNA-ID (mR), Target ID, Target annotation, the free energy of the thermodynamic ensemble (FE), the transcripts per million under normal conditions (tpm-N) or heat stress (tpm-H), the log 2 change (log2) values and the false discovery rate( fdr) value

The targets of the SL2.40ch12_12524 miRNA code for a wound-responsive protein, an eIF4A related ATP-dependent RNA helicase, a DNA repair protein rad5 and the cell wall modifying enzyme glucan endo-1 3-beta-glucosidase (Table 7). SL2.40ch09_6940 miRNA is predicted to target genes coding for a cell wall related prolyl 4-hydroxylase, glycosyltransferase, gene coding for a putative component of the CCR4-NOT complex involved in mRNA deadenylation [67] and protein similar to the human adiponectin receptor 2 (Table 7).

In contrast to the two 21 nt miRNAs identified as heat responsive in post meiotic stage, in mature pollen we found two 24 nt miRNAs from which one was induced and the other one was suppressed by heat (Fig. 6). The stress inducible SL2.40ch03_8525 miRNA was predicted to target gene coding for a cell wall pectinesterase, two genes annotated to dolichyldiphosphatase and dolichyl-diphosphooligosaccharide-protein glycosyltransferase subunit 2 involved in *N*-glycosylation, an oligopeptidase, the GTP-binding protein YqeH and a transcription factor jumonji domain-containing protein. SL2.40ch11_457 miRNA that showed a 5-fold down-regulation in heat-stressed mature pollen and is predicted to target several genes including a dTDP-4-dehydrorhamnose reductase, the nuclear protein localization 4 orthologue (Npl4) and the orthologue of *Arabidopsis* CHL27 (AT3G56940) which codes for a Mg-protoporphyrin IX monomethyl ester (MPE) cyclase involved in chloroplast development [68].

### qPCR validation of heat-responsive novel miRNAs and snoRNAs

**Fig. 7:**
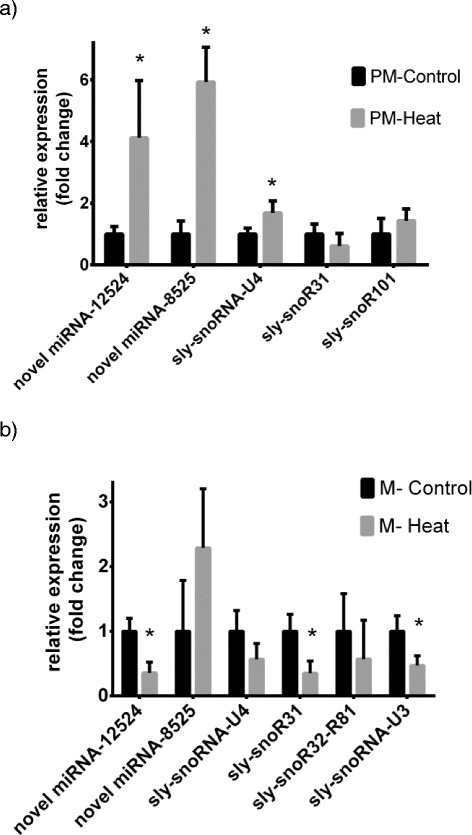
qPCR validation of heat-responsive small RNAs. Relative expression of novel miRNAs and snoRNAs in post-meiotic (**a**) and mature (**b**) pollen stages under control and heat conditions. Data presented as Mean + Standard Error of Mean (SEM). *P < 0.05. (PM – post-meiotic, M – mature)

To validate the identified heat-responsive small RNAs, we performed sncRNA-specific real-time PCR on 4 novel miRNAs and 6 snoRNAs. The differential expression of 2 miRNAs and 5 snoRNAs have been confirmed by qPCR (Fig. 7). Expression of novel miRNAs (SL2.40ch12_12524 and SL2.40ch03_8525) significantly increased under heat conditions at post-meiotic stage. Expression of SL2.40ch12_12524 miRNA significantly decreased in mature stage. These qPCR results correlate with the sequencing data.

The data for miRNAs and their targets identified in this study we also organized in an online database with a web interface (http://cab.unina.it/mirna-pollen/). It provides an access to the expression profiles of known and novel miRNAs investigated under heat and control conditions in three pollen developmental stages, and the possibility of investigating the miRNA target GO annotation using query by “*GO ID”*, “*GO description”* or “*Gene function*”. Cross-linked to a *Gbrowse* Genome browser [69] interface visualizing the miRNA tracks along the genome with the possibility of overlapping them with the ITAG2.3 gene annotation and infernals [70], Expressed Sequence Tag (EST) and Tentative Consensus (TC) tracks from 20 *Solanaceae* species [71], three Unigene collections from *SGN* [70], *PlantGDb* [72] and *Dana-Farber*; and the three pre-processed gene expression collections from different tomato genotypes (*S. lycopersicum cv. Heinz and cv. Ailsa Craig, S. pimpinellifolium*) [73] allow further genome wide investigations and comparative genomics.

## Discussion

### Small ncRNAs involved in pollen development

During heat stress, there is a general inhibition of transcription and translation and the selective induction of genes and synthesis of proteins mainly related to the protection of cellular molecules and structures [74]. Upon return to physiological conditions, the cellular homeostasis is re-established by the re-initiation of the housekeeping functions, which is marked by changes in transcriptome and proteome. Pollen development is characterized by transcriptional and translational reprogramming to facilitate the successful production of male gametes from somatic lineages [19]. In this essential process genes involved in small RNA pathways as well as diverse classes of sncRNAs play fundamental role affecting the transcriptional and translational dynamics characterizing the individual developmental stages [75]. In addition, sncRNAs are known regulators of plant HS response and genetic manipulation of specific small RNAs like miRNAs and ta-siRNAs (trans-acting siRNAs) has led to alterations in thermotolerance [76, 77]. Considering the sensitivity of pollen to heat stress and the little knowledge on the regulatory mechanisms underlying the response of this tissue to elevated temperatures, we analyzed the small RNA species present in three distinct stages of control and heat stressed tomato pollen. We were able to identify and classify sncRNAs, determine their abundance and computationally predict their putative targets.

In general in our pollen sequencing libraries we observed high fraction of singletons which have been shown to constitute a primary source of transposable elements [63]. Multiply mapped reads are the second source of TEs. 21–24-nt siRNAs are generated from TEs, tandem repeats, or exogenous sources like RNA viruses and transgenes. Arabidopsis pollen 21 nt siRNAs specifically derive from TEs [18] and can probably correspond to our tomato 22 nt sncRNAs which we observed as altered by comparing developmental stages of the pollen and under the heat in post-meiotic and mature pollen. In Arabidopsis TE-derived 21 nt siRNAs play crucial role during pollen development and have been reported to be translocated from the vegetative nucleus into the sperm cells to reinforce TE silencing [18, 19]. This process is important for pollen biogenesis and setting up unique epigenetic landscapes in gametes which influences the gamete fate. Further we observed 24 nt long RNAs that supposed to be TE-derived 24 nt hc-siRNAs (heterochromatic-siRNA). In our results, we showed that this fraction of multiple-mapped 24 nt hc-siRNAs altered significantly during pollen development and in line with the literature in our case most of 24 nt TE siRNAs are lost from mature pollen. It conceivably confirms the role of TE-derived sncRNAs in tomato pollen development and under heat stress.

We identified 425 tRNAs, seven snoRNAs, as well as 558 miRNAs both constitutively and differentially expressed under the applied stress conditions in three pollen stages. We observed a gradual change from mostly 24 nt sncRNAs in tetrads, equally distributed 24 and 22 nt sncRNAs in post-meiotic pollen and mostly 22 nT sncRNAs in mature pollen (Fig. 1). The shift in size of sncRNAs from 24 nt in pre-mature pollen stages to 22 nt in mature pollen has been also observed in rice pollen [9]. The shorter 21 nt siRNAs are preceding in *Arabidopsis* mature pollen, while 24 nt sRNAs are reduced [18]. Changes in the size of sncRNAs might be related to a shift of abundance of siRNAs and miRNAs, because these sncRNAs perform distinct functions. For example 24 nt siRNAs are involved in transcriptional gene silencing by modulating DNA and histone modifications, in contrast to 21 nt siRNAs and miRNAs that regulate their targets via mRNA degradation and translational repression [66]. Thus, the alterations in sncRNA distribution represented by the alteration of the size distributions might be relevant for pollen development and the underlying regulatory control mechanisms of this process. These mechanisms are required in particular pollen developmental time points such as transcriptional regulation in meiotic and post meiotic stages and post-transcriptional regulation in mature binucleate pollen [78]. Interestingly, we observed a decrease in the fraction of 22-nt sncRNAs in post-meiotic and mature pollen subjected to high temperature stress which might suggest increased breakdown or decreased production of particular sncRNAs due to the stress conditions.

Worth mentioning, we found a large fraction of reads mapped only once on tomato genome annotated as singletons. Although the interpretation on singletons has to be taken with some caution as the discovery and classification depends on the sequencing depth within an experiment, singletons are discussed as source of transposable elements (TE)-related siRNAs, which are thought to derive from both strands [18]. On the one hand, it has been proposed the reactivation of TE in mature pollen leads to suppression of these siRNAs [18]. On the other hand, accumulation of TE-siRNAs can lead to their activation in vegetative cells which can target gene silencing in gametes [18]. Thus, the observation of such a large fraction of singletons (54 % of all reads) documents an importance of TE-siRNAs for pollen development and function.

### snoRNAs in the regulation of heat stress response in pollen

Many of the sncRNAs classified as miRNAs, tRNAs, and snoRNAs were found to be differentially expressed in response to heat stress in particular pollen stages. Among those, the U1, U3, and U4 snoRNAs are differentially expressed in different pollen developmental stages after heat stress application (Table 2). snoRNAs are involved in modification and processing of ribosomal RNA (rRNA) and thus, heat stress induced changes in snoRNAs abundance would correlate with subsequent alterations in ribosome function [79]. An altered ribosome function would be consistent with the observed alterations of the tRNAs (Fig. 4). Alternatively, snoRNAs associated with small nuclear ribonucleoproteins (snRNP) are involved in splicing of premature mRNA transcripts [80, 81]. On the one hand, alternative splicing is enhanced under heat stress as exemplified for the moss *Physcomitrella patens* [82]. On the other hand, pollen appears to exhibit a different splicing pattern compared to seedlings as shown for *Arabidopsis thaliana* [83]. These observations together with the fact that plant pre-snoRNA processing does not require splicing [79] favor snoRNAs as one major regulator for protein synthesis and in manifesting proteome diversity in pollen in response to environmental changes.

### Heat stress induced-tRNAs in pollen

Another important class of molecules are tRNAs and tRNA-derived RNA fragments (tRFs). tRNAs are essential components of translation machinery and are involved in developmental and stress responses [84]. tRNA cleavage controls the tRNA halves abundance in a developmentally-regulated manner in the bacterium *Streptomyces coelicolor* [85], while production of tRNA-halves is a conserved response to oxidative stress in eukaryotes including plant cells [86]. We observed that the abundance of many tRNAs changed in response to heat stress often in a stage-specific manner. The Gln- and Val-tRNAs increased under heat in post-meiotic pollen, Ala- and Phe-tRNA increased under heat in mature pollen, while the abundance of Glu-, and Gly- and in lower rate Gln- and Pro-tRNAs were found to be decreased in mature pollen (Fig. 4; Tables 3 and 4). These observations can point to specific changes of the amino acid usage under stress conditions during pollen biogenesis. On the one hand, this might be related to changes in proteome under stress conditions, on the other hand to alteration of the amino acid synthesis pathways. Indeed, in some cases the capacity to accumulate proline has been correlated with stress tolerance [87–91]. Similarly a transient decrease of glutamine, proline, histidine, and tyrosine in the developing phase of caryopsis in response to high temperature was observed [92]. Lastly, the synthesis of Ala and Val has been found to increase under heat shock by 1.6- and 5.0-fold, respectively [93]. This correlates with the global increase of Ala-tRNA and the increase of Val-tRNA in post-meiotic pollen (Table 3).

### miRNAs in pollen do not control the transcript abundance in response to heat stress

miRNAs play important roles in plant development and stress responses [94, 95]. They are also involved in male germ cell development and reprogramming [96] although the global transcript abundance of miRNAs in pollen remained to be established (Fig. 3). Remarkably, most of the here identified miRNA species in tomato pollen are not differentially expressed under heat. GO term analysis of the miRNA targets demonstrated that the miRNAs are involved in the regulation of proteins involved in various processes (Fig. 5).

Four of the newly assigned miRNAs were affected by heat stress. With respect to the condition used in this study we conclude that these miRNAs play important role in the pollen thermotolerance, as being regulated in the recovery phase, during the attenuation phase of heat stress response. The targets of these miRNAs did not show differential expression in stressed samples, which might indicate possible regulation via translational repression as proposed for miRNAs and siRNAs [97]. In post-meiotic stage the heat stress up-regulated miRNA SL2.40ch12_12524 is predicted to target genes related to transcription and translation machineries such as the eIF4A related ATP-dependent RNA helicase (Solyc12g095990.1.1; *Sl*DEAD40), a Dead-box helicase protein [98]. RNA helicases, as regulators of every step of RNA metabolism, are involved in several abiotic stress responses [99]. For example, *Arabidopsis* AT1G54270, the best homologue hit of Solyc12g095990.1.1 [98], is differentially regulated by cold stress in suspension cells [100]. Similarly, Solyc12g020110.1.1 s is predicted to code for a Rad5 protein, a member of the Snf2 ATPase/helicase family [101]. Rad5 is a key component of post-replication repair machinery and mitotic recombination in *S. cerevisiae* [102]. Thus, it probably has important role in mitotic phase of pollen development especially under stress conditions. Another proposed target of SL2.40ch12_12524 is Solyc01g007950 that codes for a peroxidase. Among all proposed target genes Solyc01g007950 is the only one that is reduced in response to heat stress (by 50 %). The *Arabidopsis* Solyc01g007950 orthologue codes for a constitutively expressed lipase gene (At1g10740) [103]. Thus, a slight reduction might have significant physiological consequences for pollen development and thermotolerance.

The SL2.40ch09_6940 miRNA is exclusively expressed under heat stress in the post-meiotic stage. This miRNA has been predicted to target CCR4-NOT, which is required for efficient transcription of plant microRNA and protein coding genes [104] as well as the pre-miRNA processing. In *Saccharomyces cerevisiae,* the Ccr4–Not protein complex interacts with general stress transcriptional regulators Msn2/4 [104, 105] and with Skn7 [106]. Skn7 interacts with Hsf1 *in vivo* and is required for the induction of heat shock genes [107], which links CCR4-NOT function to heat stress response regulation.

Both miRNAs target genes related to cell wall biosynthesis and modification such as a glucan endo-1 3-beta-glucosidase, a glycosyltransferase and a prolyl 4-hydroxylase (P4H). Members of the P4H gene family were recently shown to be involved in tomato leaf growth by affecting cell expansion and division [108]. Moreover, P4Hs as well as their major substrates, hydroxyproline-rich glycoproteins, have been shown to be responsive to various abiotic stresses [109, 110]. Other genes found here as targets of two stress induced miRNAs have been shown to be regulated by heat stress including the Magnesium protoporhyrin IX and laccase [111, 112]. Therefore, we conclude that the two miRNAs identified are involved in the regulation of pollen heat stress response, although we suggest a translational rather than a transcriptional impact.

In mature stage we identified one miRNA induced and a second reduced after heat stress applicaton. This suggests a divergent regulatory role of the miRNAs in stress responses in male gametophyte. Again, the mRNA levels of the target genes remained unchanged in response to stress. A putative target of the stress-induced SL2.40ch03_8525 miRNA is a pectinesterase coding gene, which supports a miRNA-based control of cell wall related proteins. A second target codes for a prolyl oligopeptidase (POP). Ectopic expression of rice OsPOP5 in *E. coli* increased tolerance to several abiotic stresses including high temperature [113] supporting a function of the identified miRNAs in the regulation of pollen thermotolerance.

## Conclusion

sncRNAs are important regulators of developmental and stress response mechanisms. We show that heat stress alters the sncRNA expression landscape, especially in post-meiotic and mature stages of male gametophyte development. We observed significant changes in the levels of specific sncRNA species. The alterations in tRNAs related to specific amino acids might have an important consequence for different cellular processes and needs to be further examined. We also identified four novel heat stress responsive miRNAs with possible tissue- and stage-specific functions. The fact, that the novel miRNAs have not been previously reported for other tomato tissues conceivably indicate the pollen specific expression, although this needs to be experimentally proven. Quantitative analysis of their targets point to the regulation in a non mRNA-degradation pathway. Some targets have not been identified so far as heat stress responsive but their predicted functions indicate possible involvement in mechanisms of pollen thermotolerance and development. Of course, the mode of action of these miRNAs on their targets as well as the specific functions of the targets in the stress response needs to be further investigated. Further the elucidation of the cross-function between different types of pollen sncRNAs in heat-stress response will contribute to our understanding of the pollen thermotolerance mechanism in general and extend the strategy for finding biomarkers usable in future breeding strategies directed to the production of heat-tolerant cultivars.

### Availability of supporting data

The data sets supporting the results of this article are available in the Array Express repository, accession E-MTAB-3830.

## Additional files

Additional file 1: Figure S1. Overview of the heat stress conditions used in the study. (A) Pictograph showing the stress regime used for plant treatment as described in material and methods. (B) Representative photos of flower buds corresponding to different pollen stages. (C) Photos of pollen cells from different developmental stages, stained with the Alexander dye. Positive staining indicates viable pollen. (D) Bright field image of pollen at tetrad stage and fluorescent image of post-meiotic and mature pollen stained with DAPI, showing the single and double nuclei, respectively. (DOCX 2811 kb) Additional file 2: Table S5. Primer sequences of selected sncRNAs for qPCR validation. (DOCX 13 kb) Additional file 3: Table S1. List of known miRNAs detected in three pollen libraries [114-120]. (XLSX 18 kb) Additional file 4: Table S2. Novel putative miRNAs in post-meiotic pollen with their chromosomal position, mapping coordinates, sequence of the predicted miRNA precursor, star sequence, and mature sequence. (XLSX 71 kb) Additional file 5: Table S3. Novel putative miRNAs in mature pollen with their chromosomal position, mapping coordinates, sequence of the predicted miRNA precursor, star sequence, and mature sequence. (XLSX 23 kb) Additional file 6: Table S4. Predicted targets of miRNAs and GO annotation. (XLSX 732 kb)
