# Supplementary material for: Identification of novel small ncRNAs in pollen of tomato
Source: BMC Genomics. 2015 Sep 18;16(1):714. doi: 10.1186/s12864-015-1901-x (PMC4575465; doi:10.1186/s12864-015-1901-x)
Supplement: Additional file 1: Figure S1. — Overview of the heat stress conditions used in the study. (A) Pictograph showing the stress regime used for plant treatment as described in material and methods. (B) Representative photos of flower buds corresponding to different pollen stages. (C) Photos of pollen cells from different developmental stages, stained with the Alexander dye. Positive staining indicates viable pollen. (D) Bright field image of pollen at tetrad stage and fluorescent image of post-meiotic and mature pollen stained with DAPI, showing the single and double nuclei, respectively. (DOCX 2811 kb) [file 12864_2015_1901_MOESM1_ESM.docx]

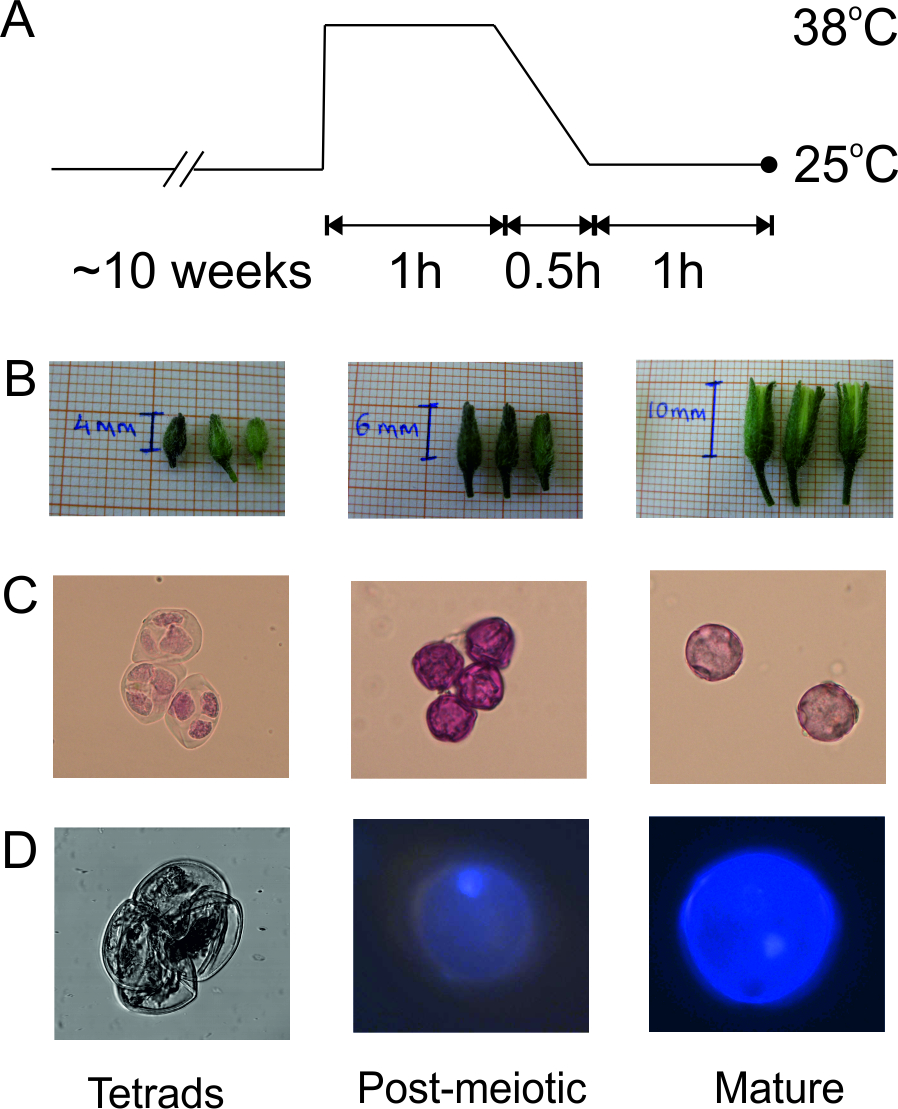


Additional file 1: Figure S1. Overview of the heat stress conditions used in the study. (A) Pictograph showing the stress regime used for plant treatment as described in material and methods. (B) Representative photos of flower buds corresponding to different pollen stages. (C) Photos of pollen cells from different developmental stages, stained with the Alexander dye. Positive staining indicates viable pollen. (D) Bright field image of pollen at tetrad stage and fluorescent image of post meiotic and mature pollen stained with DAPI, showing the single and double nuclei, respectively.
