# Supplementary material for: Identification of novel small ncRNAs in pollen of tomato
Source: BMC Genomics. 2015 Sep 18;16(1):714. doi: 10.1186/s12864-015-1901-x (PMC4575465; doi:10.1186/s12864-015-1901-x)
Supplement: Additional file 2: Table S5. — Primer sequences of selected sncRNAs for qPCR validation. (DOCX 13 kb) [file 12864_2015_1901_MOESM2_ESM.docx]

Additional file 2: Table S5: Primer sequences of selected sncRNAs for qPCR validation. (F – forward, R – reverse)

| Primer name | Primer sequence 5 ˋ-3ˋ |
| --- | --- |
| novel_miRNA-12524 | F CGCAGTCATCTCCCTACA |
|  | R CCAGTTTTTTTTTTTTTTTACTTGCCT |
| novel_miRNA_8525 | F CGCAGATTGTTGACATAAGTACC |
|  | R GTCCAGTTTTTTTTTTTTTTTGCAG |
| sly-snoRNA-U4 | F GAAAGGGCTTCCCTTGCGGGTA |
|  | R CAGTTTTTTTTTTTTTTTGGGCTCT |
| sly-snoR101 | F GCAGTGCATATGATGGCT |
|  | R CAGGTCCAGTTTTTTTTTTTTTTTGTA |
| sly-snoR31 | F CAGCGCAGTCAAATGATGAT |
|  | R CAGGTCCAGTTTTTTTTTTTTTTTGTA |
| sly-snoR32_R81 | F GCGCAGGAAAATGATGAATATAAG |
|  | R GGTCCAGTTTTTTTTTTTTTTTCTC |
| sly-snoRNA-U3 | F GGTCTCATGGCTGTCTGA |
|  | R TCCAGTTTTTTTTTTTTTTTGTCTGT |
| 5S_rRNA | F GGGATGCGATCATACCAG |
|  | R GGTCCAGTTTTTTTTTTTTTTTAGTG |

|  |  |
| --- | --- |
